# Telemedicine and health disparities: Association between the area deprivation index and primary care telemedicine utilization during the COVID-19 pandemic

**DOI:** 10.1017/cts.2023.580

**Published:** 2023-07-10

**Authors:** Mina Ostovari, Zugui Zhang, Vishal Patel, Claudine Jurkovitz

**Affiliations:** Christiana Care Health Services Inc., Wilmington, DE, USA

**Keywords:** Telemedicine, primary care, chronic disease, area deprivation index (ADI), health disparities, COVID-19

## Abstract

**Introduction:**

The rapid implementation of telemedicine during the COVID-19 pandemic may have exacerbated the existing health disparities. This study investigated the association between the area deprivation index (ADI), which serves as a measure of socioeconomic deprivation within a geographic area, and the utilization of telemedicine in primary care.

**Methods:**

The study data source was electronic health records. The study population consisted of patients with at least one primary care visit between March 2020 and December 2021. The primary outcome of interest was the visit modality (office, phone, and video). The exposure of interest was the ADI score grouped into quartiles (one to four, with one being the least deprived). The confounders included patient sociodemographic characteristics (e.g., age, gender, race, ethnicity, insurance coverage, marital status). We utilized generalized estimating equations to compare the utilization of telemedicine visits with office visits, as well as phone visits with video visits.

**Results:**

The study population included 41,583 patients with 127,165 office visits, 39,484 phone visits, and 20,268 video visits. Compared to patients in less disadvantaged neighborhoods (ADI quartile = one), patients in more disadvantaged neighborhoods (ADI = two, three, or four) had higher odds of using phone visits vs office visits, lower odds of using video visits vs office visits, and higher odds of using phone visits vs video visits.

**Conclusions:**

Patients who resided in socioeconomically disadvantaged neighborhoods mainly relied on phone consultations for telemedicine visits with their primary care provider. Patient-level interventions are essential for achieving equitable access to digital healthcare, particularly for low-income individuals.

## Introduction

During the COVID-19 pandemic, health systems rapidly implemented and expanded telemedicine to ensure continued access to care [1– 3]. In addition to screening and monitoring patients with COVID-19 symptoms [4], telemedicine was utilized for chronic disease management [5–7]. Telemedicine can be clinically as effective as in-person visits for some conditions, including diabetes [8,9]. However, for other conditions, such as heart failure, studies have shown mixed results [10]. Furthermore, there is limited evidence regarding the effectiveness of telemedicine in different clinical settings, such as primary care vs. specialty care [11].

While telemedicine has the potential to enhance access to care [12], several studies have indicated that it may also exacerbate the already existing health disparities [13]. As telemedicine becomes increasingly integral to healthcare delivery, it is crucial to identify factors that may hinder patients’ ability to benefit from this technology. Patient socioeconomic status impacts their access to care and health outcomes [14]. Individuals living in disadvantaged areas may face health risks associated with the characteristics of their neighborhoods [15,16]. The area deprivation index (ADI) serves as a multi-dimensional measure that represents the socioeconomic deprivation of a geographic area [17].

ADI was initially developed by the U.S. Federal Government over three decades ago and later modified by Kind and Her research team at the University of Wisconsin-Madison [18]. The ADI measure, composed of 17 census variables that describe the socioeconomic disadvantages of a neighborhood based on education, income/employment, housing status, and household characteristics [17], captures social factors not collected in the electronic health records (EHRs) [19].

This study presents a longitudinal analysis of primary care telemedicine vs. office (in-person) visits utilization for patients with diabetes, hypertension, heart failure, and/or chronic obstructive pulmonary disease (COPD). These conditions were selected due to their high prevalence among the health system patient population and the feasibility of managing them through home telemonitoring programs [20].

While prior studies have examined patient sociodemographic factors associated with telemedicine utilization, such as age, gender, race, income, and insurance [21–23], we focused on ADI as this variable represents patient’s socioeconomic status and the potential impact of the neighborhood on access to care. The study findings will inform health systems strategies to advance equity in digital healthcare delivery.

## Materials and methods

### Study population & data source

This study was approved by the Christiana Care Institutional Review Board. Christiana Care Health System is one of the largest health systems in the mid-Atlantic region that serves all of Delaware, parts of Pennsylvania, Maryland, and New Jersey. The study population included patients with a diagnosis of diabetes, heart failure, hypertension, and/or COPD with at least one billed primary care ambulatory visit (phone, video, and/or office) between March 2020 and December 2021. Patient demographic and clinical characteristics were extracted from the EHR. Patient ADI scores defined at the census tracts level were obtained from the already computed 2020 ADI scores in Neighborhood Atlas [18]. We used national ADI measures as it provides better generalizability [24]. To determine patient’s ADI score, we extracted patients’ addresses at their index visit, geocoded the addresses using ArcGIS 10.8, and mapped the geocodes to the census tracts.

### Telemedicine visits

Since the onset of the COVID-19 pandemic in March 2020, the study health system provided primary care services both through telemedicine (video or phone) and office (in-person) visits. For telemedicine visits, patients were required to create an account on the health system patient portal and log in before their scheduled appointment. Prior to the telemedicine visit, a healthcare professional (such as a physician/nurse assistant) would contact the patient via phone to address any questions or concerns, collect biometrics (weight and blood pressure), and help the patient log in to the patient portal account. A phone visit would replace a video visit based on patient preferences or if they had difficulty using the patient portal.

### Statistical analysis

The analytical dataset included patient telemedicine (video or phone) and office visits recorded between March 2020 and December 2021. The outcome of interest was the visit modality (office, phone, or video). The exposure of interest was ADI, which was split into quartiles (one to four, with one being the least disadvantaged). We compared the ADI quartile of the index visit with the ADI quartile of the last visit during the study period and found minimal fluctuations. The last ADI quartile was bigger than the first ADI quartile for less than 2% of patients (*N* = 715) and smaller than the first ADI quartile for about 2% of patients (*N* = 1138). The confounders included patient age, gender (male/female), race (White, Black/African American, or other (Asian, American Indian or Alaska, Pacific Islander, other race, and decline/unavailable)), ethnicity (Hispanic or Latino, non-Hispanic or Latino, and unknown/declined), marital status (married, legal partner, divorced, separated, widowed, single, or unknown), the primary insurance provider (Medicare, commercial, Medicaid, or Self-pay), a diagnosis of diabetes, hypertension, heart failure, and/or COPD. In the EHR, missing patient characteristics are shown as unknown/unavailable. For confounder variables, we included missing values as a category. As 97% of patients’ primary language was recorded as English, we did not include language as a confounder.

We defined an additional indicator variable, “Time,” to account for the month in which the visit occurred. The Time variable was assigned a value of 1 for visits that occurred in March 2020, with subsequent months incrementing the value by one unit. We examined the interaction of Time and ADI to assess any potential modifying impacts.

We employed generalized estimating equations (GEE) to examine the association between ADI and visit modality. GEE is a robust statistical method that allows accounting for repeated outcomes [25]. The majority of patients in our dataset had multiple visits, including office visits and/or telemedicine (video/phone) consultations (main outcome). Each patient was assigned a unique identifier (Patient ID), which was used as the repeated factor in the GEE model. We defined an indicator variable, Visit Order, to capture the sequence of the visits for each patient.

Four distinct statistical models were constructed for the analysis. In the first model, the outcome had three categories: office, phone, and video. In the second model, an interaction term was introduced between the ADI quartile and the Time variable to assess whether Time influenced the association between ADI and the outcome. The third model focused on telemedicine visits (phone vs. video). In the fourth model, we added an interaction term between the ADI quartile and Time to examine whether Time modified the impact of ADI quartile on the utilization of phone vs. video visits. To illustrate the interactions, we generated interaction plots depicting the predicted probabilities for each visit type. A *p*-value ≤ 0.05 was considered statistically significant. All statistical analyses were done in SAS software v 9.4 (SAS Institute, Carry NC).

## Results

### Telemedicine and office visit utilization

**Figure 1.**
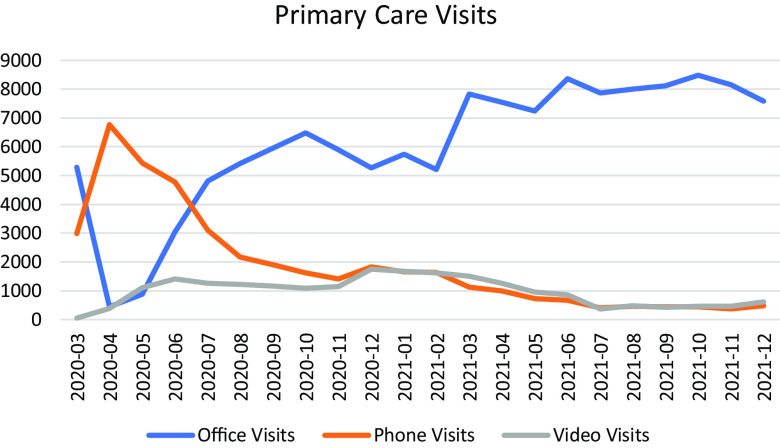
Study population office, phone, and video visits utilization during the study period.

During the study period, the study population had 127,165 office visits, 39,484 phone visits, and 20,268 video visits. Between March and June 2020, there was a higher number of phone visits compared to office and video visits. Starting in June 2020, there was a noticeable upward trend in the number of office visits, while the number of phone and video visits gradually decreased. These trends remained relatively stable through the rest of the study period. Fig. 1 represents the utilization of office, phone, and video visits for the study population between March 2020 and December 2021.

### Study population

There were 43,683 patients with at least one office, phone, and/or video visit between March 2020 and December 2021. We were able to obtain the already calculated 2020 ADIs for 95% of the study population (*N* = 41,583) from the Neighborhood Atlas[18]. For the rest of the population (*N* = 2100), the ADIs were missing. We categorized the ADI scores into quartiles to distinguish between the most and the least disadvantaged neighborhoods and were unable to include patients with missing ADI scores in the analysis. We checked the characteristics of patients (age, gender, race, and ethnicity) with missing ADIs but did not identify any specific patterns. Our final study population included 41,583 individuals, including 22,206 female and 19,377 male patients, with an average age of 62 (SD = 14.2).

Table 1 shows the study population demographics and their distribution based on ADI quartiles. ADI quartile one represents the least disadvantaged neighborhoods and ADI quartile four represents the most disadvantaged neighborhoods. The majority of patients were in ADI quartile two (41%), followed by ADI quartile three (26%), ADI quartile one (25%), and ADI quartile four (7%).

**Table 1.** Distribution of patient characteristics by area deprivation index (ADI) quartiles.

Study population, *N* = 41,583	ADI *Q* = 1(ADI ≤ 25), *N* = 10,540	ADI *Q* = 2 (25<ADI ≤ 50), *N* = 17,292	ADI *Q* = 3 (50<ADI ≤ 75), *N* = 10,676	ADI *Q* = 4 (ADI > 75), *N* = 3075
Gender				
Female, 22,206 (53%)	5051 (48%)	9174 (53%)	6145(58%)	1836 (60%)
Male, 19,377 (47%)	5489 (52%)	8118 (47%)	4531(42%)	1239 (40%)
Ethnicity				
Hispanic or Latino, 1394 (3%)	206 (2%)	472 (3%)	529 (5%)	187 (6%)
Non-Hispanic or Latin, 39,132 (94%)	10,049 (95%)	16,321 (94%)	9911 (93%)	2851 (93%)
Unknown/declined, 1057 (3%)	285 (3%)	499 (3%)	236 (2%)	37 (1%)
Race				
White, 27,440 (66%)	8523 (80.9%)	12,254 (70.9%)	5956 (55.8%)	707 (23%)
Black/African American, 11,824 (28.4%)	1366 (13%)	4146 (24%)	4139 (38.8%)	2173 (70.7%)
Asian, 1096 (2.6%)	458 (4.3%)	459 (2.7%)	148 (1.4%)	31 (1%)
American Indian or Alaska Native, 122 (0.3%)	36 (0.3%)	36 (0.2%)	40 (0.4%)	10 (0.3%)
Pacific Islander, 25 (0.1%)	4 (0.04%)	14 (0.1%)	7 (0.1%)	0
Other Race, 790 (1.9%)	97 (0.9%)	260 (1.5%)	300 (2.8%)	133 (4%)
Decline/unavailable, 286 (0.7%)	56 (0.5%)	123 (0.7%)	86 (0.8%)	21 (0.7%)
Marital Status				
Divorced, 3789 (9.1%)	638 (6.1%)	1579 (9.1%)	1211 (11.3%)	361 (11.7%)
Legally separated, 458 (1.1%)	48 (0.5%)	134 (0.8%)	181 (1.7%)	95 (3.1%)
Widowed, 4807 (11.6%)	1067 (10.1%)	2032 (11.8%)	1358 (12.7%)	350 (11.4%)
Married, 23,233 (55.9%)	7570 (71.8%)	10,222 (59.1%)	4630 (43.4%)	811 (26.4%)
Life partner, 88 (0.2%)	25 (0.2%)	37 (0.2%)	22 (0.2%)	4 (0.1%)
Single, 9037 (21.7%)	1159 (11%)	3212 (18.6%)	3227 (30.2%)	1439 (46.8%)
Unknown, 171 (0.4%)	33 (0.3%)	76 (0.4%)	47 (0.4%)	15 (0.5%)
Type of Insurance				
Commercial, 17,153 (41%)	4759 (45%)	7601 (44%)	4051 (38%)	742 (24%)
Medicare, 20,202 (49%)	5362 (51%)	8422 (49%)	4967 (46%)	1451 (47%)
Medicaid, 3620 (9%)	328 (3%)	1046 (6%)	1446 (14%)	800 (26%)
Self-pay, 608 (1%)	91 (1%)	223 (1%)	212 (2%)	82 (3%)
COPD, 3730 (9%)	717 (7%)	1547 (9%)	1102 (10%)	364 (12%)
Diabetes, 14,397 (35%)	2991 (28%)	5970 (34%)	4202 (39%)	1234 (40%)
Heart Failure, 1671 (4%)	295 (3%)	614 (4%)	545 (5%)	217 (7%)
Hypertension, 36,789 (88%)	9469 (90%)	15,358 (89%)	9301 (87%)	2661 (87%)

In the first column, the denominator is the total number of patients. In the second to fourth columns, the denominator is the number of individuals in each ADI quartile.

In addition to patient demographics, we examined the frequency of patient biometrics (BMI, systolic, and diastolic blood pressure) recorded in telemedicine and office visits. Patient BMI was recorded in 102,206 office visits (80% of office visits), 1802 phone visits (5% of phone visits), and 2530 video visits (12% of video visits). Systolic blood pressure was recorded in 122,708 office visits (96%), 3,676 phone visits (9%), and 4271 video visits (21%). Diastolic blood pressure was recorded in 122,622 office visits (96%), 3671 phone visits (9%), and 4265 video visits (21%). We also examined the final diagnosis recorded for office, phone, and video visits. The most common final diagnoses were the same for office, phone, and video visits and included primary hypertension (I10), disorders of lipoprotein metabolism and other lipidemia (E78), and Type 2 diabetes mellitus (E11).

### GEE results comparing the utilization of video and phone visits to office visits

**Figure 2.**
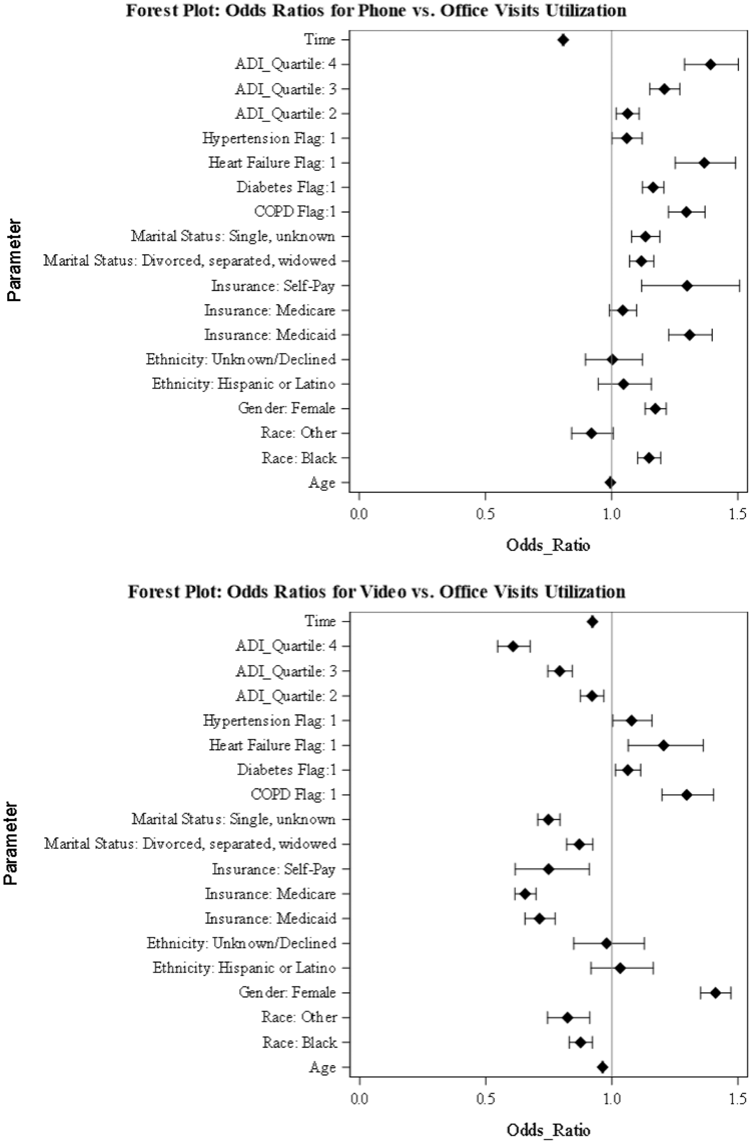
Generalized estimating equations results. The figures show the odds ratios of predictors for phone visits vs. office visits utilization and video visits vs. office visits utilization. ADI = area deprivation index, COPD = chronic obstructive pulmonary disease.

We generated a GEE model to compare the utilization of video and phone visits vs. office visits. Fig. 2 presents the odds ratios and confidence intervals for the predictors, including ADI quartiles and the Time variable (please see the supplement material document for additional information).

As shown in Fig. 2, individuals in ADI quartiles two, three, or four had lowers odds of using video visits vs. office visits and higher odds of using phone visits vs. office visits compared to the reference group (ADI quartile one).

Increasing age was associated with lower utilization of video visits (aOR, 0.963 [95% CI, 0.961–0.965]) and phone visits (aOR, 0.995 [95% CI, 0.993–0.997]) vs. office visits. When compared to white patients, Black or African American patients had lower odds of using video visits (aOR, 0.876 [95% CI, 0.831–0.922]) vs office visits, but higher odds of using phone visits (aOR, 1.148 [95% CI, 1.103–1.194]). Compared to patients with commercial insurance, patients with Medicaid or Self-pay had lower odds of using video visits vs. office visits (aOR, 0.713 [95% CI, 0.656–0.775]), (aOR, 0.749 [95% CI, 0.617–0.910]), but higher odds of using phone visits vs. office visits (aOR, 1.309 [95% CI, 1.226–1.398]), (aOR, 1.299 [95% CI, 1.119–1.507]).

**Figure 3.**
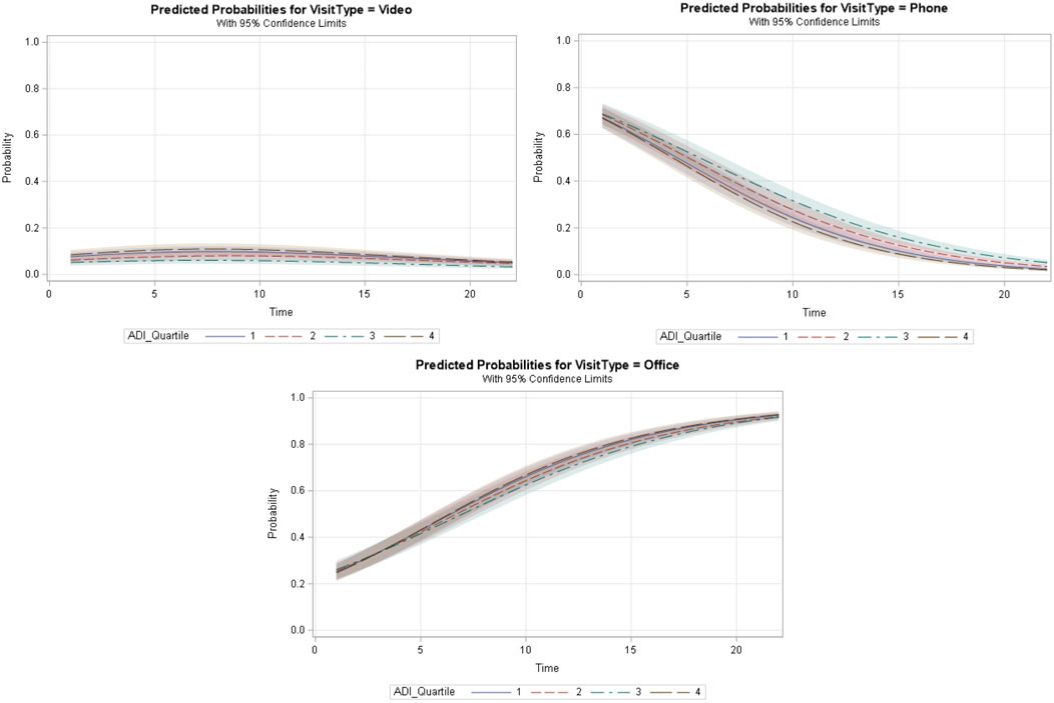
Graphs represent the interaction between area deprivation index (ADI) quartiles and Time variables and the impact on the utilization of video, phone, and office visits. The y-axis shows the predicted probability of the visit type for each type of visit (video, phone, office). The x-axis shows the Time variable (in months). Fit computed at age = 62.9, race = Black/African American, gender = female, ethnicity = non-Hispanic or Latino, insurance = Medicare, marital status = divorced/separated/widowed.

To examine whether the Time variable modified the impact of ADI quartiles on the utilization of video and phone visits vs. office visits, we added an interaction term between the ADI quartile and Time (Table 2 in the supplement material). Based on the GEE results, the interaction was significant between Time and ADI quartile two, for phone vs. office utilization (aOR, 1.011 [95% CI, 1.002–1.021]), ADI quartile three, and video vs. office utilization (aOR, 1.011 [95% CI, 1.004–1.018]), ADI quartile three, and phone vs. office utilization (aOR, 1.028 [95% CI, 1.018–1.039]), and ADI quartile four, and phone v. office utilization (aOR, 1.049 [95% CI, 1.035–1.063]). Fig. 3 shows the interaction curves generated at each level of the ADI quartiles.

The probability of utilizing office visits increased for all ADI levels with time, whereas the probability of having a phone visit decreased consistently. While the probability of using video visits increased slightly during the first five months, it remained almost constant for the rest of the study period.

## GEE results comparing the utilization of phone vs video visits

**Figure 4.**
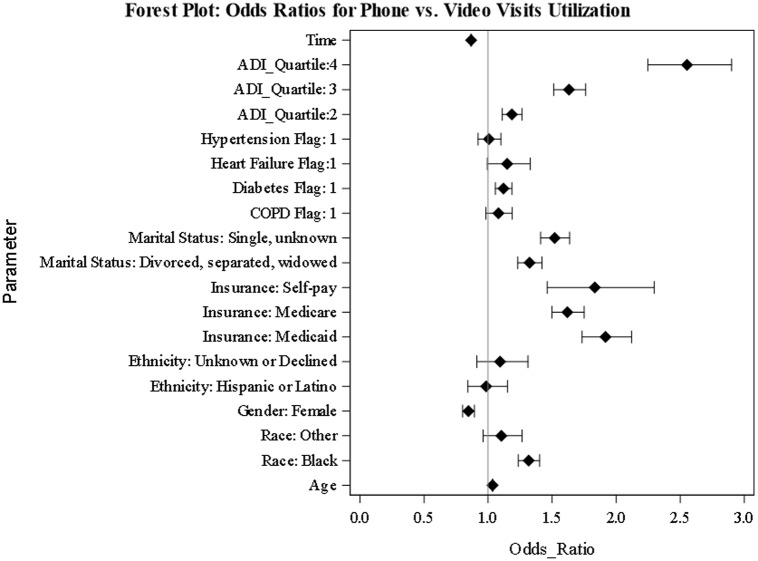
Generalized estimating equations results. The figure shows the odds ratios for phone visits vs. video visits utilization. ADI = area deprivation index, COPD = chronic obstructive pulmonary disease.

We used GEE to model the utilization of phone vs. video visits during the study period. We also examined the interaction between ADI quartiles and Time in a separate model. Fig. 4 represents the odds ratios for the predictors.

As shown in Fig. 4, patients who lived in ADI quartiles two, three, or four had higher odds of using phone visits vs. video visits compared to individuals residing in ADI quartile one (the reference group). Similar to the previous analysis, Black/African Americans had higher odds of using phone visits compared to white patients (aOR, 1.317 [95% CI, 1.236–1.403]). Compared to patients with commercial insurance coverage, patients with Medicaid or Self-pay had higher odds of using phone vs. video visits (aOR, 1.917 [95% CI, 1.734–2.120]), (aOR, 1.833 [95% CI, 1.462–2.297]), respectively.

**Figure 5.**
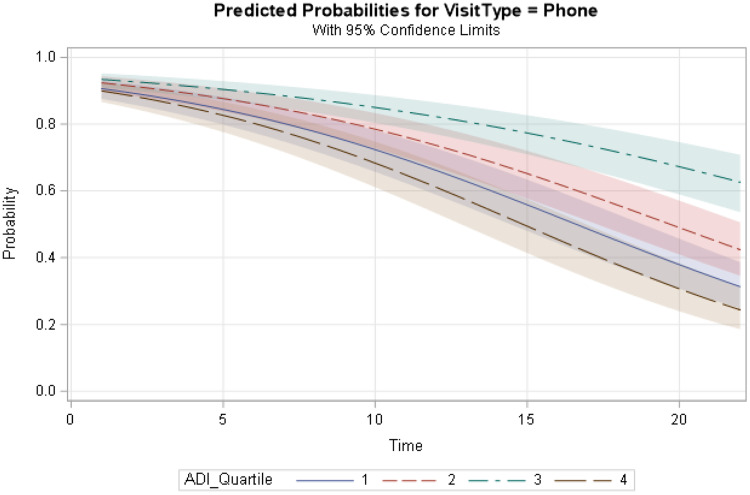
Graph represents the interaction between area deprivation index (ADI) quartiles and time variable and the impact on phone vs. video utilization. The *y*-axis shows the predicted probability of phone visits. The *x*-axis shows the Time variable (in months). Fit computed at age = 62.9, race = Black/African American, gender = female, ethnicity = non-Hispanic or Latino, insurance = Medicare, marital status = divorced/separated/widowed.

The interaction between ADI quartiles and Time was significant for all levels of ADI quartiles. Fig. 5 shows the interaction plot between ADI quartiles and Time. For all patients, regardless of their ADI category, the probability of utilizing phone visits decreased with time.

## Discussion

In this study, we examined the association between ADI and telemedicine utilization during the COVID-19 pandemic in patients with hypertension, diabetes, heart failure, and COPD. We stratified the study population into ADI quartiles and compared telemedicine (phone or video) vs. office visits utilization as well as phone vs. video visits utilization.

Similar to another study [26], patients from disadvantaged areas (patients in ADI quartile two, three, or four) were more likely to rely on phone consultations for telemedicine visits compared to patients from the least disadvantaged category (ADI quartile one). Despite efforts to expand telemedicine services in the health system, the utilization of video visits did not increase during the study period, even among individuals from the less disadvantaged neighborhoods (ADI quartile one). By examining the characteristics of patients less likely to utilize telemedicine, health systems can customize their strategies to meet patient-specific needs and promote equitable care. It is important to recognize that certain patients may prefer in-person visits for various reasons, such as lack of trust or dissatisfaction with telemedicine services[27].

Similar to other studies [28–32], Black or African Americans, and individuals covered by Medicaid were more likely to use phone visits vs. video visits/office visits. The lower rate of video visits utilization among patients could be attributed to various factors, such as limited digital literacy, inadequate infrastructure in their neighborhood, or language barriers [33–35]. Recognizing and addressing the specific needs of the patient population is crucial for health systems, as the presence of clinical and technical infrastructure may not be sufficient to improve telemedicine utilization [36].

Our findings also revealed that increasing age was associated with lower odds of using telemedicine (phone or video visits) vs. office visits as well as higher odds of using phone visits vs. video visits. These results are comparable to those of other studies [37,38]. Limited digital literacy and trust issues often pose obstacles for older adults when it comes to adopting technology [39,40]. Reducing the complexity of telemedicine platforms and providing educational resources, specifically designed for the elderly, could help improve the usage of telemedicine services among these individuals [41,42].

This study has some limitations that should be considered when interpreting the results. The study population was limited to one health system, which may limit the generalizability of the results. Although we used national ADI scores to enhance generalizability, the association between ADI and telemedicine utilization may vary in other regions of the U.S. Therefore, conducting similar studies in health systems across different geographical areas could be valuable. We mainly focused on service utilization and did not investigate health outcomes. In future studies, we will examine the impact of telemedicine utilization on condition-specific patient outcomes.

## Conclusions

Examining the utilization of telemedicine (phone/video) vs. office visits and phone vs. video visits revealed that individuals who lived in disadvantaged neighborhoods mainly relied on phone consultations for telemedicine services. To enhance equitable access to telemedicine, health systems may need to implement additional strategies that improve access to all aspects of telemedicine, particularly video visits and patient portals.

## Supplementary material

For supplementary material accompanying this paper visit https://doi.org/10.1017/cts.2023.580. click here to view supplementary material
