# Supplementary material for: Telemedicine and health disparities: Association between the area deprivation index and primary care telemedicine utilization during the COVID-19 pandemic
Source: J Clin Transl Sci. 2023 Jul 10;7(1):e168. doi: 10.1017/cts.2023.580 (PMC10425871; doi:10.1017/cts.2023.580)
Supplement: Supplementary file 1 [file S2059866123005800sup001.docx]

**Table 1**. Generalized Estimating Equations (GEE) Results Comparing the Utilization of Video and Phone Visits vs. Office Visits

| **Effect** | | **Visit Type** | **Point Estimates** | **Odds Ratio 95% CI** | | **P-value** |
| --- | --- | --- | --- | --- | --- | --- |
| ADI_ Quartile | 2 | Video | 0.921 | 0.875 | 0.968 | 0.001 |
| ADI_ Quartile | 2 | Phone | 1.063 | 1.018 | 1.109 | 0.006 |
| ADI_ Quartile | 3 | Video | 0.793 | 0.746 | 0.843 | <.0001 |
| ADI_ Quartile | 3 | Phone | 1.209 | 1.151 | 1.270 | <.0001 |
| ADI_ Quartile | 4 | Video | 0.608 | 0.547 | 0.676 | <.0001 |
| ADI_ Quartile | 4 | Phone | 1.392 | 1.289 | 1.502 | <.0001 |
| Time* | | Video | 0.923 | 0.920 | 0.925 | <.0001 |
| Time* | | Phone | 0.808 | 0.805 | 0.811 | <.0001 |
| Age | | Video | 0.963 | 0.961 | 0.965 | <.0001 |
| Age | | Phone | 0.995 | 0.993 | 0.997 | <.0001 |
| Race | Black/African American | Video | 0.876 | 0.831 | 0.922 | <.0001 |
| Race | Black/African American | Phone | 1.148 | 1.103 | 1.194 | <.0001 |
| Race | Other | Video | 0.824 | 0.745 | 0.912 | 0.0002 |
| Race | Other | Phone | 0.920 | 0.842 | 1.006 | 0.0676 |
| Gender | Female | Video | 1.411 | 1.352 | 1.472 | <.0001 |
| Gender | Female | Phone | 1.173 | 1.133 | 1.216 | <.0001 |
| Ethnicity | Hispanic or Latino | Video | 1.033 | 0.917 | 1.164 | 0.596 |
| Ethnicity | Hispanic or Latino | Phone | 1.047 | 0.947 | 1.157 | 0.367 |
| Ethnicity | Unknown/Declined | Video | 0.979 | 0.849 | 1.129 | 0.771 |
| Ethnicity | Unknown/Declined | Phone | 1.003 | 0.897 | 1.122 | 0.960 |
| Insurance | Medicaid | Video | 0.713 | 0.656 | 0.775 | <.0001 |
| Insurance | Medicaid | Phone | 1.309 | 1.226 | 1.398 | <.0001 |
| Insurance | Medicare | Video | 0.656 | 0.616 | 0.699 | <.0001 |
| Insurance | Medicare | Phone | 1.044 | 0.991 | 1.099 | 0.104 |
| Insurance | Self-pay | Video | 0.749 | 0.617 | 0.910 | 0.004 |
| Insurance | Self-pay | Phone | 1.299 | 1.119 | 1.507 | 0.0006 |
| Marital status | Divorced/Separated/Widowed | Video | 0.871 | 0.821 | 0.924 | <.0001 |
| Marital status | Divorced/Separated/Widowed | Phone | 1.118 | 1.071 | 1.167 | <.0001 |
| Marital status | Single/ Unknown | Video | 0.748 | 0.706 | 0.794 | <.0001 |
| Marital status | Single/ Unknown | Phone | 1.134 | 1.079 | 1.191 | <.0001 |
| COPD | 1 | Video | 1.297 | 1.199 | 1.403 | <.0001 |
| COPD | 1 | Phone | 1.296 | 1.225 | 1.370 | <.0001 |
| Diabetes | 1 | Video | 1.063 | 1.014 | 1.114 | 0.012 |
| Diabetes | 1 | Phone | 1.164 | 1.122 | 1.207 | <.0001 |
| Heart Failure | 1 | Video | 1.205 | 1.065 | 1.363 | 0.003 |
| Heart Failure | 1 | Phone | 1.367 | 1.252 | 1.491 | <.0001 |
| Hypertension | 1 | Video | 1.078 | 1.004 | 1.159 | 0.039 |
| Hypertension | 1 | Phone | 1.060 | 1.001 | 1.121 | 0.045 |
| **References** Race: White, Ethnicity: non-Hispanic or Latino, Gender: Male, Insurance: Commercial, Marital status: Partner/Married  *Time: time variable is calculated in month. Quasi information criterion (QIC)= 262003.184 | | | | | | |

**Table 2**. Generalized Estimating Equations (GEE) Results Comparing the Utilization of Video and Phone Visits vs. Office Visits with ADI Quartiles and Time Interaction

| Parameter | | Visit Type | Point Estimate | Odds Ratio 95% CI | | Pr > \|Z\| |
| --- | --- | --- | --- | --- | --- | --- |
| Time*ADI_Quartile | 2 | Video | 1.003 | 0.996 | 1.009 | 0.427 |
| Time*ADI_ Quartile | 2 | Phone | 1.011 | 1.002 | 1.021 | 0.019 |
| Time*ADI_ Quartile | 3 | Video | 1.011 | 1.004 | 1.018 | 0.003 |
| Time*ADI_Quartile | 3 | Phone | 1.028 | 1.018 | 1.039 | <.0001 |
| Time*ADI_ Quartile | 4 | Video | 1.002 | 0.990 | 1.014 | 0.711 |
| Time*ADI_ Quartile | 4 | Phone | 1.049 | 1.035 | 1.063 | <.0001 |
| ADI_ Quartile | 2 | Video | 0.892 | 0.818 | 0.972 | 0.009 |
| ADI_ Quartile | 2 | Phone | 0.975 | 0.904 | 1.050 | 0.501 |
| ADI_ Quartile | 3 | Video | 0.696 | 0.630 | 0.770 | <.0001 |
| ADI_ Quartile | 3 | Phone | 0.962 | 0.887 | 1.044 | 0.352 |
| ADI_ Quartile | 4 | Video | 0.580 | 0.492 | 0.683 | <.0001 |
| ADI_ Quartile | 4 | Phone | 0.927 | 0.825 | 1.041 | 0.199 |
| Time |  | Video | 0.919 | 0.914 | 0.923 | <.0001 |
| Time |  | Phone | 0.794 | 0.787 | 0.800 | <.0001 |
| Age | | Video | 0.963 | 0.961 | 0.965 | <.0001 |
| Age | | Phone | 0.995 | 0.993 | 0.997 | <.0001 |
| Race | Black/African American | Video | 0.875 | 0.831 | 0.922 | <.0001 |
| Race | Black/African American | Phone | 1.146 | 1.102 | 1.193 | <.0001 |
| Race | Other | Video | 0.825 | 0.745 | 0.912 | 0.0002 |
| Race | Other | Phone | 0.924 | 0.845 | 1.010 | 0.0817 |
| Gender | Female | Video | 1.411 | 1.352 | 1.472 | <.0001 |
| Gender | Female | Phone | 1.173 | 1.132 | 1.215 | <.0001 |
| Ethnicity | Hispanic or Latino | Video | 1.033 | 0.916 | 1.164 | 0.597 |
| Ethnicity | Hispanic or Latino | Phone | 1.046 | 0.947 | 1.155 | 0.3804 |
| Ethnicity | Unknown/Declined | Video | 0.979 | 0.849 | 1.129 | 0.7712 |
| Ethnicity | Unknown/Declined | Phone | 1.003 | 0.896 | 1.123 | 0.9556 |
| Insurance | Medicaid | Video | 0.712 | 0.654 | 0.774 | <.0001 |
| Insurance | Medicaid | Phone | 1.301 | 1.219 | 1.389 | <.0001 |
| Insurance | Medicare | Video | 0.656 | 0.616 | 0.699 | <.0001 |
| Insurance | Medicare | Phone | 1.045 | 0.993 | 1.101 | 0.092 |
| Insurance | Self-pay | Video | 0.749 | 0.617 | 0.910 | 0.004 |
| Insurance | Self-pay | Phone | 1.302 | 1.123 | 1.509 | 0.0005 |
| Marital status | Divorced/Separated/Widowed | Video | 0.748 | 0.705 | 0.794 | <.0001 |
| Marital status | Divorced/Separated/Widowed | Phone | 1.119 | 1.072 | 1.168 | <.0001 |
| Marital status | Single/ Unknown | Video | 0.748 | 0.705 | 0.794 | <.0001 |
| Marital status | Single/ Unknown | Phone | 1.134 | 1.079 | 1.191 | <.0001 |
| COPD | 1 | Video | 1.298 | 1.200 | 1.403 | <.0001 |
| COPD | 1 | Phone | 1.295 | 1.225 | 1.369 | <.0001 |
| Diabetes | 1 | Video | 1.062 | 1.013 | 1.114 | 0.012 |
| Diabetes | 1 | Phone | 1.164 | 1.122 | 1.206 | <.0001 |
| Heart Failure | 1 | Video | 1.204 | 1.064 | 1.362 | 0.003 |
| Heart Failure | 1 | Phone | 1.365 | 1.252 | 1.488 | <.0001 |
| Hypertension | 1 | Video | 1.078 | 1.004 | 1.158 | 0.040 |
| Hypertension | 1 | Phone | 1.060 | 1.001 | 1.121 | 0.045 |
| **References**  Race: White, Ethnicity: non-Hispanic or Latino, Gender: Male, Insurance: Commercial, Marital status: Partner/Married  *Time: time variable is calculated in month. QIC= 261881.443 | | | | | | |

**Table 3**. Generalized Estimating Equations (GEE) Results Comparing the Utilization of Phone Visits vs. Video Visits

| Parameter | | Point Estimate | Odds Ratio 95% CI | | Pr > \|Z\| |
| --- | --- | --- | --- | --- | --- |
| ADI_ Quartile | 2 | 1.186 | 1.111 | 1.265 | <.0001 |
| ADI_ Quartile | 3 | 1.632 | 1.512 | 1.762 | <.0001 |
| ADI_ Quartile | 4 | 2.553 | 2.247 | 2.901 | <.0001 |
| Time |  | 0.868 | 0.865 | 0.872 | <.0001 |
| Age | | 1.035 | 1.032 | 1.038 | <.0001 |
| Race | Black/African American | 1.317 | 1.236 | 1.403 | <.0001 |
| Race | Other | 1.104 | 0.962 | 1.266 | 0.159 |
| Gender | Female | 0.847 | 0.802 | 0.894 | <.0001 |
| Ethnicity | Hispanic or Latino | 0.985 | 0.842 | 1.152 | 0.846 |
| Ethnicity | Unknown/Declined | 1.094 | 0.912 | 1.312 | 0.335 |
| Insurance | Medicaid | 1.917 | 1.734 | 2.120 | <.0001 |
| Insurance | Medicare | 1.619 | 1.499 | 1.750 | <.0001 |
| Insurance | Self-pay | 1.833 | 1.462 | 2.297 | <.0001 |
| Marital status | Divorced/Separated/Widowed | 1.324 | 1.232 | 1.421 | <.0001 |
| Marital status | Single/ Unknown | 1.520 | 1.411 | 1.637 | <.0001 |
| COPD | 1 | 1.08 | 0.98 | 1.19 | 0.11 |
| Diabetes | 1 | 1.12 | 1.06 | 1.19 | 0.00 |
| Heart Failure | 1 | 1.15 | 0.99 | 1.33 | 0.06 |
| Hypertension | 1 | 1.01 | 0.92 | 1.10 | 0.88 |
| **References**  Race: White, Ethnicity: non-Hispanic or Latino, Gender: Male, Insurance: Commercial, Marital status: Partner/Married  Modeling probability that visit type= Phone  *Time: time variable is calculated in month. QIC=63456.5808 | | | | | |

**Table 4**. Generalized Estimating Equations (GEE) Results Comparing the Utilization of Phone Visits vs. Video Visits with ADI and Time Interaction

| Parameter | | Point Estimate | Odds Ratio 95% CI | | Pr > \|Z\| |
| --- | --- | --- | --- | --- | --- |
| Time*ADI_Quartile | 2 | 1.013 | 1.001 | 1.025 | 0.033 |
| Time*ADI_ Quartile | 3 | 1.025 | 1.013 | 1.038 | <.0001 |
| Time*ADI_ Quartile | 4 | 1.058 | 1.041 | 1.076 | <.0001 |
| ADI_ Quartile | 2 | 1.067 | 0.960 | 1.185 | 0.230 |
| ADI_ Quartile | 3 | 1.317 | 1.171 | 1.482 | <.0001 |
| ADI_ Quartile | 4 | 1.491 | 1.243 | 1.789 | <.0001 |
| Time |  | 0.854 | 0.845 | 0.862 | <.0001 |
| Age | | 1.035 | 1.032 | 1.038 | <.0001 |
| Race | Black/African American | 1.316 | 1.236 | 1.402 | <.0001 |
| Race | Other | 1.108 | 0.966 | 1.271 | 0.144 |
| Gender | Female | 0.847 | 0.802 | 0.894 | <.0001 |
| Ethnicity | Hispanic or Latino | 0.985 | 0.842 | 1.151 | 0.846 |
| Ethnicity | Unknown/Declined | 1.094 | 0.912 | 1.313 | 0.334 |
| Insurance | Medicaid | 1.916 | 1.735 | 2.117 | <.0001 |
| Insurance | Medicare | 1.628 | 1.506 | 1.759 | <.0001 |
| Insurance | Self-pay | 1.843 | 1.473 | 2.306 | <.0001 |
| Marital status | Divorced/Separated/Widowed | 1.326 | 1.235 | 1.425 | <.0001 |
| Marital status | Single/ Unknown | 1.519 | 1.411 | 1.636 | <.0001 |
| COPD | 1 | 1.079 | 0.981 | 1.186 | 0.118 |
| Diabetes | 1 | 1.118 | 1.056 | 1.184 | 0.000 |
| Heart Failure | 1 | 1.149 | 0.994 | 1.327 | 0.060 |
| Hypertension Flag | 1 | 1.004 | 0.918 | 1.096 | 0.938 |
| **References**  Race: White, Ethnicity: non-Hispanic or Latino, Gender: Male, Insurance: Commercial, Marital status: Partner/Married  *Time: time variable is calculated in month. QIC=63397.5803 | | | | | |

**Examining the False Discovery Rate**

We adjusted the p-value using the Bonferroni adjustment to control the false discovery rate (FDR) for models comparing video and phone vs. office visits and phone vs. video visit. Controlling the FDR helps reduce the likelihood of falsely identifying significant effects when comparing multiple factors or conducting multiple tests. By adjusting the significance threshold or p-value cutoff, the FDR correction aims to strike a balance between controlling the number of false discoveries and not missing true discoveries. The following tables show the Bonferroni adjustment results for each predictor. By comparing the Pr > |z| column to the Adj P column, we can see that the p-values are adjusted upwards; in this case, there is no change in the conclusions.

**Phone and Video vs. Office Utilization**

**Table 5**. Differences of ADI_Quartile Least Squares Means

Adjustment for Multiple Comparisons: Bonferroni

| Visit Type | ADI_Quartile | _ADI_Quartile | Estimate | Standard Error | z Value | Pr > \|z\| | Adj P |
| --- | --- | --- | --- | --- | --- | --- | --- |
| Video | 1 | 2 | 0.149 | 0.028 | 5.330 | <.0001 | <.0001 |
| Video | 1 | 3 | 0.414 | 0.052 | 8.020 | <.0001 | <.0001 |
| Video | 1 | 4 | -0.083 | 0.026 | -3.210 | 0.001 | 0.008 |
| Video | 2 | 3 | 0.265 | 0.052 | 5.070 | <.0001 | <.0001 |
| Video | 2 | 4 | -0.232 | 0.031 | -7.430 | <.0001 | <.0001 |
| Video | 3 | 4 | -0.497 | 0.054 | -9.190 | <.0001 | <.0001 |
| Phone | 1 | 2 | -0.129 | 0.021 | -6.050 | <.0001 | <.0001 |
| Phone | 1 | 3 | -0.270 | 0.036 | -7.410 | <.0001 | <.0001 |
| Phone | 1 | 4 | 0.061 | 0.022 | 2.770 | 0.006 | 0.034 |
| Phone | 2 | 3 | -0.140 | 0.037 | -3.810 | 0.000 | 0.001 |
| Phone | 2 | 4 | 0.190 | 0.025 | 7.600 | <.0001 | <.0001 |
| Phone | 3 | 4 | 0.330 | 0.039 | 8.470 | <.0001 | <.0001 |

**Table 6**. Differences of Race Least Squares Means

Adjustment for Multiple Comparisons: Bonferroni

| Visit Type | Race | _Race | Estimate | Standard Error | z Value | Pr > \|z\| | Adj P |
| --- | --- | --- | --- | --- | --- | --- | --- |
| Video | Black | Other | 0.061 | 0.055 | 1.110 | 0.268 | 0.805 |
| Video | Black | White | -0.133 | 0.026 | -5.020 | <.0001 | <.0001 |
| Video | Other | White | -0.194 | 0.052 | -3.750 | 0.000 | 0.001 |
| Phone | Black | Other | 0.221 | 0.048 | 4.620 | <.0001 | <.0001 |
| Phone | Black | White | 0.138 | 0.020 | 6.740 | <.0001 | <.0001 |
| Phone | Other | White | -0.083 | 0.045 | -1.830 | 0.068 | 0.203 |

**Table 7**. Differences of Gender Least Squares Means

Adjustment for Multiple Comparisons: Bonferroni

| Visit Type | Gender | _Gender | Estimate | Standard Error | z Value | Pr > \|z\| | Adj P |
| --- | --- | --- | --- | --- | --- | --- | --- |
| Video | Female | Male | 0.344 | 0.022 | 15.890 | <.0001 | <.0001 |
| Phone | Female | Male | 0.160 | 0.018 | 8.890 | <.0001 | <.0001 |

**Table 8**. Differences of Ethnicity Least Squares Means

Adjustment for Multiple Comparisons: Bonferroni

| Visit Type | Ethnicity | _Ethnicity | Estimate | Standard Error | z Value | Pr > \|z\| | Adj P |
| --- | --- | --- | --- | --- | --- | --- | --- |
| Video | Hispanic or Latino | Non-Hispanic or Latino | 0.054 | 0.094 | 0.570 | 0.567 | 1 |
| Video | Hispanic or Latino | Unknown/Declined | 0.032 | 0.061 | 0.530 | 0.596 | 1 |
| Video | Non-Hispanic or Latino | Unknown/Declined | -0.021 | 0.073 | -0.290 | 0.771 | 1 |
| Phone | Hispanic or Latino | Non-Hispanic or Latino | 0.043 | 0.075 | 0.570 | 0.566 | 1 |
| Phone | Hispanic or Latino | Unknown/Declined | 0.046 | 0.051 | 0.900 | 0.367 | 1 |
| Phone | Non-Hispanic or | Unknown/Declined | 0.003 | 0.057 | 0.050 | 0.960 | 1 |

**Table 9**. Differences of Insurance Least Squares Means

Adjustment for Multiple Comparisons: Bonferroni

| Visit Type | Insurance | Insurance | Estimate | Standard Error | z Value | Pr > \|z\| | Adj P |
| --- | --- | --- | --- | --- | --- | --- | --- |
| Video | Commercial | Medicaid | 0.083 | 0.050 | 1.660 | 0.096 | 0.577 |
| Video | Commercial | Medicare | -0.050 | 0.105 | -0.470 | 0.636 | 1.000 |
| Video | Commercial | Self-Pay | -0.338 | 0.043 | -7.900 | <.0001 | <.0001 |
| Video | Medicaid | Medicare | -0.132 | 0.102 | -1.300 | 0.193 | 1.000 |
| Video | Medicaid | Self-Pay | -0.421 | 0.032 | -13.150 | <.0001 | <.0001 |
| Video | Medicare | Self-Pay | -0.289 | 0.099 | -2.920 | 0.004 | 0.021 |
| Phone | Commercial | Medicaid | 0.226 | 0.040 | 5.670 | <.0001 | <.0001 |
| Phone | Commercial | Medicare | 0.008 | 0.080 | 0.100 | 0.922 | 1.000 |
| Phone | Commercial | Self-Pay | 0.269 | 0.034 | 8.010 | <.0001 | <.0001 |
| Phone | Medicaid | Medicare | -0.219 | 0.078 | -2.790 | 0.005 | 0.032 |
| Phone | Medicaid | Self-Pay | 0.043 | 0.026 | 1.630 | 0.104 | 0.621 |
| Phone | Medicare | Self-Pay | 0.262 | 0.076 | 3.440 | 0.001 | 0.003 |

**Table 10**. Differences of Marital Status Least Squares Means

Adjustment for Multiple Comparisons: Bonferroni

| Visit Type | Marital status | _Marital status | Estimate | Standard Error | z Value | Pr > \|z\| | Adj P |
| --- | --- | --- | --- | --- | --- | --- | --- |
| Video | Divorced/separated/widowed | Married/partner | 0.152 | 0.038 | 4.020 | <.0001 | 0.0002 |
| Video | Divorced/separated/widowed | Single/unknown | -0.138 | 0.030 | -4.580 | <.0001 | <.0001 |
| Video | Married/partner | Single/unknown | -0.290 | 0.030 | -9.630 | <.0001 | <.0001 |
| Phone | Divorced/separated/widowed | Married/partner | -0.014 | 0.029 | -0.480 | 0.629 | 1 |
| Phone | Divorced/separated/widowed | Single/unknown | 0.112 | 0.022 | 5.130 | <.0001 | <.0001 |
| Phone | Married/partner | Single/unknown | 0.126 | 0.025 | 4.980 | <.0001 | <.0001 |

**Table 11**. Differences of COPD Least Squares Means

Adjustment for Multiple Comparisons: Bonferroni

| Visit Type | COPD | _COPD | Estimate | Standard Error | z Value | Pr > \|z\| | Adj P |
| --- | --- | --- | --- | --- | --- | --- | --- |
| Video | 0 | 1 | 0.260 | 0.039 | 6.51 | <.0001 | <.0001 |
| Phone | 0 | 1 | 0.259 | 0.029 | 9.05 | <.0001 | <.0001 |

**Table 12**. Differences of Diabetes Least Squares Means

Adjustment for Multiple Comparisons: Bonferroni

| Visit Type | Diabetes | _Diabetes | Estimate | Standard Error | z Value | Pr > \|z\| | Adj P |
| --- | --- | --- | --- | --- | --- | --- | --- |
| Video | 0 | 1 | 0.061 | 0.024 | 2.530 | 0.012 | 0.012 |
| Phone | 0 | 1 | 0.152 | 0.018 | 8.210 | <.0001 | <.0001 |

**Table 13**. Differences of Heart Failure Least Squares Means

Adjustment for Multiple Comparisons: Bonferroni

| Visit Type | Heart Failure | _Heart Failure | Estimate | Standard Error | z Value | Pr > \|z\| | Adj P |
| --- | --- | --- | --- | --- | --- | --- | --- |
| Video | 0 | 1 | 0.186 | 0.063 | 2.950 | 0.003 | 0.003 |
| Phone | 0 | 1 | 0.312 | 0.044 | 7.030 | <.0001 | <.0001 |

**Table 14.** Differences of Hypertension Least Squares Means

Adjustment for Multiple Comparisons: Bonferroni

| Visit Type | Hypertension | _Hypertension | Estimate | Standard Error | z Value | Pr > \|z\| | Adj P |
| --- | --- | --- | --- | --- | --- | --- | --- |
| Video | 0 | 1 | 0.075 | 0.037 | 2.060 | 0.039 | 0.039 |
| Phone | 0 | 1 | 0.058 | 0.029 | 2.000 | 0.045 | 0.045 |

**Phone vs. Video Analysis**

**Table 15**. Differences of ADI_Quartile Least Squares Means

Adjustment for Multiple Comparisons: Bonferroni

| ADI_Quartile | _ADI_Quartile | Estimate | Standard Error | z Value | Pr > \|z\| | Adj P |
| --- | --- | --- | --- | --- | --- | --- |
| 1 | 2 | -0.319 | 0.034 | -9.32 | <.0001 | <.0001 |
| 1 | 3 | -0.767 | 0.061 | -12.44 | <.0001 | <.0001 |
| 1 | 4 | 0.170 | 0.033 | 5.12 | <.0001 | <.0001 |
| 2 | 3 | -0.447 | 0.062 | -7.2 | <.0001 | <.0001 |
| 2 | 4 | 0.49 | 0.039 | 12.55 | <.0001 | <.0001 |
| 3 | 4 | 0.937 | 0.065 | 14.37 | <.0001 | <.0001 |

**Table 16**. Differences of Race Least Squares Means

Adjustment for Multiple Comparisons: Bonferroni

| Race | _Race | Estimate | Standard Error | z Value | Pr > \|z\| | Adj P |
| --- | --- | --- | --- | --- | --- | --- |
| Black | Other | 0.177 | 0.073 | 2.42 | 0.0155 | 0.0465 |
| Black | White | 0.275 | 0.032 | 8.54 | <.0001 | <.0001 |
| Other | White | 0.098 | 0.070 | 1.41 | 0.1591 | 0.4772 |

**Table 17**. Differences of Gender Least Squares Means

Adjustment for Multiple Comparisons: Bonferroni

| Gender | _Gender | Estimate | Standard Error | z Value | Pr > \|z\| | Adj P |
| --- | --- | --- | --- | --- | --- | --- |
| Female | Male | -0.166 | 0.028 | -6 | <.0001 | <.0001 |

**Table 18**. Differences of Ethnicity Least Squares Means

Adjustment for Multiple Comparisons: Bonferroni

| Ethnicity | _Ethnicity | Estimate | Standard Error | z Value | Pr > \|z\| | Adj P |
| --- | --- | --- | --- | --- | --- | --- |
| Hispanic or Latino | Non-Hispanic or Latino | -0.105 | 0.120 | -0.87 | 0.382 | 1 |
| Hispanic or Latino | Unknown/Declined | -0.016 | 0.080 | -0.19 | 0.846 | 1 |
| Non-Hispanic or Latino | Unknown/Declined | 0.089 | 0.093 | 0.96 | 0.335 | 1 |

**Table 19**. Differences of Insurance Least Squares Means

Adjustment for Multiple Comparisons: Bonferroni

| Insurance | _Insurance | Estimate | Standard Error | z Value | Pr > \|z\| | Adj P |
| --- | --- | --- | --- | --- | --- | --- |
| Commercial | Medicaid | 0.169 | 0.061 | 2.78 | 0.0055 | 0.033 |
| Commercial | Medicare | 0.045 | 0.123 | 0.37 | 0.7129 | 1 |
| Commercial | Self-Pay | 0.651 | 0.051 | 12.7 | <.0001 | <.0001 |
| Medicaid | Medicare | -0.124 | 0.119 | -1.04 | 0.3002 | 1 |
| Medicaid | Self-Pay | 0.482 | 0.039 | 12.16 | <.0001 | <.0001 |
| Medicare | Self-Pay | 0.606 | 0.115 | 5.26 | <.0001 | <.0001 |

**Table 20**. Differences of Marital status Least Squares Means

Adjustment for Multiple Comparisons: Bonferroni

| Marital status | _Marital status | Estimate | Standard Error | z Value | Pr > \|z\| | Adj P |
| --- | --- | --- | --- | --- | --- | --- |
| Divorced/separated/widowed | Married/partner | -0.139 | 0.046 | -3.04 | 0.0024 | 0.0071 |
| Divorced/separated/widowed | Single/unknown | 0.280 | 0.036 | 7.7 | <.0001 | <.0001 |
| Married/partner | Single/unknown | 0.419 | 0.038 | 11.06 | <.0001 | <.0001 |

**Table 21**. Differences of COPD Least Squares Means

Adjustment for Multiple Comparisons: Bonferroni

| COPD | _COPD | Estimate | Standard Error | z Value | Pr > \|z\| | Adj P |
| --- | --- | --- | --- | --- | --- | --- |
| 0 | 1 | 0.078 | 0.049 | 1.6 | 0.1091 | 0.1091 |

**Table 22**. Differences of Diabetes Least Squares Means

Adjustment for Multiple Comparisons: Bonferroni

| Diabetes | _Diabetes | Estimate | Standard Error | z Value | Pr > \|z\| | Adj P |
| --- | --- | --- | --- | --- | --- | --- |
| 0 | 1 | 0.113 | 0.029 | 3.85 | 0.0001 | 0.0001 |

**Table 23**. Differences of Heart FailureLeast Squares Means

Adjustment for Multiple Comparisons: Bonferroni

| Heart Failure | _Heart Failure | Estimate | Standard Error | z Value | Pr > \|z\| | Adj P |
| --- | --- | --- | --- | --- | --- | --- |
| 0 | 1 | 0.139 | 0.074 | 1.88 | 0.0605 | 0.0605 |

**Table 24**. Differences of Hypertension Least Squares Means

Adjustment for Multiple Comparisons: Bonferroni

| Hypertension | _Hypertension | Estimate | Standard Error | z Value | Pr > \|z\| | Adj P |
| --- | --- | --- | --- | --- | --- | --- |
| 0 | 1 | 0.0068 | 0.045 | 0.15 | 0.8802 | 0.8802 |
